# Upper gastrointestinal bleeding in a patient with depression receiving selective serotonin reuptake inhibitor therapy

**DOI:** 10.4103/0253-7613.48881

**Published:** 2009-02

**Authors:** Deepak Kumar, Tanuj Saaraswat, S.N. Sengupta, Saurabh Mehrotra

**Affiliations:** Department of Psychiatry, Institute of Human Behaviour and Allied Sciences (IHBAS), Dilshad Garden, Delhi, India

**Keywords:** Depression, risk factors, selective serotonin reuptake inhibitor, upper gastrointestinal bleeding

## Abstract

**Background:**

Serotonin plays an important role in the normal clotting phenomenon and is released by platelets. Platelets are dependent on a serotonin transporter for the uptake of serotonin, as they cannot synthesize it themselves. Selective serotonin reuptake inhibitors (SSRIs) block the uptake of serotonin into platelets and can cause problems with clotting leading to bleeding.

**Aim:**

This case report highlights the occurrence of upper gastrointestinal bleeding in the index case on initiating SSRI therapy for depression and the prompt resolution of the same on its discontinuation on two separate occasions.

**Conclusion:**

SSRIs may cause upper gastrointestinal (GI) bleeding. Physicians should be aware of the same and should try to rule out previous episodes of upper GI bleed or the presence of other risk factors which might predispose to it before prescribing SSRIs; they should also warn the patients about this potential side effect. Also, the presence of thalassemia trait in the index patient deserves special attention and needs to be explored to see if it might in any way contribute in potentiating this side effect of SSRIs.

## Introduction

Serotonin is released from platelets in response to vascular injury, and promotes vasoconstriction and a change in the shape of the platelets, which leads to aggregation.[1] Platelets cannot themselves synthesize serotonin. Selective Serotonin Reuptake Inhibitors (SSRIs) inhibit the serotonin transporter, which is responsible for the uptake of serotonin into platelets. Thus, they lower intraplatelet serotonin concentration[2,3] and, at least some of them, also lower the expression of the platelet activation marker CD63 in response to thrombin receptor–activating peptide.[4]

It has thus been postulated that SSRIs would deplete platelet serotonin, leading to a reduced ability to form clots and a subsequent increase in the risk of bleeding.[2,5] This case report highlights the finding of episodes of upper gastrointestinal (GI) bleed in an inpatient on SSRI therapy for depression.

## Case Report

A 35-year-old housewife from an urban background and belonging to the middle socio-economic status reported at our hospital (a tertiary care neuro-psychiatric hospital in Northern India), with complaints of persistent-pervasive sadness of mood, depressive cognitions, suspiciousness, anxiety, irritability, and suicidal ideation for eight months and one suicidal attempt about seven months ago. The patient had had a similar episode, which started about four years prior to the current one and lasted for about two years. The patient attempted suicide twice and the symptoms remitted after administration of 12 sessions of Modified Electroconvulsive Therapy (MECTs). When the patient first reported to our hospital, she was already on a cocktail regimen of mirtazapine (30 mg/d), quitiepine (200 mg/d), duloxetine (60 mg/d), lamotrigine (100 mg/d), and buspirone (30 mg/d) from a private practitioner.

The aforesaid medications were tapered and stopped (from the outpatient department), in view of their ineffectiveness and venlafaxine was initiated and built up to a dose of 150 mg/d over a period of one week and continued. However, the patient did not show any significant improvement (on BDI rating scale) and her suicidal ideation persisted, so she was admitted for administration of MECTs. Routine investigations including thyroid profile were carried out. The hemogram showed low hemoglobin levels (suggestive of anemia) and erythrocytes being Naked Eye Single Tube Red Cell Osmotic Fragility Test (NESTROFT) positive (suggestive of Thalassemia or thalassemia trait). Further investigations (hemoglobin-electrophoresis) showed evidence of thalassemia trait (which probably was the cause of anemia). During MECTs, she developed low oxygen saturation in blood repeatedly, because of which further MECTs had to be stopped. At the same time, the patient developed hypertension with consistently elevated blood pressure (130-140 mmHg systolic and 90-100 mmHg diastolic) due to which venlafaxine was tapered and stopped and antihypertensive medication started. The blood pressure normalized over the next few days, with consistent systolic readings in the range of 120s and diastolic readings in the range of low 80s). The patient was subsequently started on the SSRI sertraline and built up to a dose of 100 mg/day.

One week after the initiation of SSRI therapy, the patient had an episode of vomiting, which consisted of about 5-7 ml of bright red blood and another episode occurred eight hours later; however, this time the vomitus had a similar amount of coffee-colored blood. Overall, the patient had five such episodes over a period of four days. In view of the temporal relation between administration of sertraline and the bleeding as well as the potential GI bleeding due to SSRIs,[5,6] sertraline was stopped in a tapering dose and the episodes of bleeding completely subsided.

The patient was shifted to dothiepin 225mg/d, but she did not show any improvement. It was decided to administer MECTs after a high-risk consent. After the administration of the 5^th^ MECT, the patient developed post-ECT confusion and so the MECTs had to be discontinued. As the depressive symptoms worsened despite TCA therapy for four weeks, it was decided to shift her to an SSRI with lower degree of serotonin reuptake inhibition as significant association between degree of serotonin reuptake inhibition and risk of abnormal bleeding has been reported.[7] Thus, escitalopram, an SSRI, with a lower degree of serotonin uptake inhibition[8] was started and built up to a dose of 20 mg/d. Six days later, she again had episodes of vomiting blood, sometimes fresh-red, but mostly coffee-colored. As the patient refused to get an upper gastrointestinal (GI) endoscopy done, an upper GI barium series was carried out and the patient was also referred for an ENT examination to rule out other causes of bleeding. Both the investigations were normal. The patient never had any instance of upper GI bleeding prior to these episodes nor was she receiving NSAIDs. Owing to these episodes of bleeding, escitalopram was tapered and stopped and duloxetine was started as monotherapy. She did not have any further episode of upper GI bleed.

## Discussion

SSRIs are commonly used in the treatment of depression because of their efficacy and a favorable safety and tolerability profile. Side effects like sexual dysfunction, nausea, diarrhea, anxiety, insomnia, sedation, nightmares and rare instances of extra pyramidal symptoms are well known. However, bleeding (GI or ecchymoses) has also been noticed and it has become a matter of avid research. There have been a few retrospective studies, in recent years, showing higher relative risk in cases as compared to controls.[9–12]

These studies have generally shown that the use of SSRIs is associated with increased incidence of upper gastrointestinal bleeding (GI) and that the SSRIs may play a causal role in it. Also, an association between the risk of bleeding and increasing affinity for the serotonin transporter has been noted in several studies and this was the reason for shifting our patient from sertraline to escitalopram.[7–10] The risk decreased to the same level as controls in past users of SSRIs, indicating that bleeding is likely to be associated with the drug rather than the illness it was prescribed for.[13] The association also holds when age, gender, and the effects of other drugs such as aspirin and NSAIDs are controlled for.

The risk is especially high in the presence of other risk factors like NSAIDs anti-platelet drugs, ulcers, erosions[8–10,12,13] or old age.[12] The association with NSAIDs has been shown to be more than additive. The mechanisms by which NSAIDs and SSRIs are associated with GI bleeding are different. NSAIDs directly damage the gastrointestinal mucosa, while SSRIs reduce the effectiveness of the normal clotting mechanism. Aspirin does both. The absolute additional risk of an upper GI bleed (requiring admission to hospital) with an SSRI prescribed alone is about one in 300 patient years, but co-prescription of SSRIs with aspirin increases the risk to one in 200 and with NSAIDs to one in 80.[10] The risk with a non-steroidal drug alone is one in 200.[14]

In the index case, although no other clear cut risk factor could be detected, the temporal relationship between the onset of SSRI therapy and bleeding, more than once, is impressive. Equally impressive is the prompt resolution of the problem with discontinuation of the therapy and hence the association between SSRI therapy and upper GI bleed seems to be quite high. The authors however acknowledge the limitations in establishing the causality. Also, literature mentions hematological side effects with other antidepressants (including TCA's and venlafaxine).[15,16]

The relationship between SSRIs and the above-mentioned risk factors has been studied, but whether the presence of thalassemia trait in the index case was also contributory to the bleeding (especially with SSRI therapy) is an unanswered question. Further studies are needed to investigate whether caution should also be exercised in prescribing SSRIs to patients of thalassemia or those in which other risk factors for bleeding like hypertension are present. Also, as no other clear-cut risk factor could be identified in the index case, the authors wonder whether any genetic predisposition might be present as well, in certain patients. Further studies should seek to inquire this aspect as well.

In view of these findings it is suggested that physicians be cautious in prescribing SSRIs and should seek to find out additional risk factors of GI bleed or past history suggestive of the same in patients who are to be prescribed SSRIs, Gastro protection (in the form of proton pump inhibitors or H2 receptor antagonists) is advised, especially when concomitantly using NSAIDS. In fact, such issues are being raised in recent years[17]. The effects of SSRIs on bleeding are not predicted by standard blood tests but are revealed by platelet-aggregation tests.[15] As these tests are not likely to be readily available in the developing countries, the physicians in these countries should exercise even more caution in finding out other risk factors for GI bleed before prescribing SSRIs.

### Statement of interest

The authors have no conflict of interest with any commercial or other associations in connection with the submitted article.
